# Mind–body therapies for sleep disturbances, depression, and anxiety in menopausal women: a systematic review and meta-analysis of randomized controlled trials

**DOI:** 10.3389/fpubh.2025.1686981

**Published:** 2025-11-18

**Authors:** Zhixin Fan, Yifan Zhang, Yankai Shu, Yongxu Zhou, Zhongzhi Zuo

**Affiliations:** 1 Graduate School of Physical Education, Myongji University, Yongin, Republic of Korea; 2 College of Physical Education, Hunan University of Science and Technology, Xiangtan, Hunan, China; 3 Graduate School of Physical Education, Kookmin University, Seoul, Republic of Korea

**Keywords:** menopause, mind–body therapies, sleep disturbances, depression, anxiety, art therapy

## Abstract

**Background:**

Menopause is frequently accompanied by sleep disturbances, depression, and anxiety, negatively affecting women’s quality of life. While hormone therapy can be effective, safety concerns highlight the need for accessible non-pharmacological options. Mind–body therapies (MBTs) have emerged as promising interventions, yet their overall efficacy remains unclear.

**Methods:**

A systematic review and meta-analysis were conducted on randomized controlled trials evaluating the effects of MBTs—such as Yoga, Mindfulness, Qigong, Art therapy, Music therapy, Dance therapy and Reiki—on sleep, depression, and anxiety in perimenopausal and postmenopausal women. Comparator groups included usual care or no-intervention controls. Eighteen studies involving 1,572 participants were identified through PubMed, Embase, Cochrane Library, and Web of Science (through October 10, 2025). Random-effects models were applied, along with subgroup, sensitivity, and publication bias analyses.

**Results:**

MBTs significantly improved sleep quality (SMD = −0.86; 95% CI: −1.24 to −0.48), reduced depression (SMD = −0.79; 95% CI: −1.18 to −0.40), and alleviated anxiety (SMD = −1.13; 95% CI: −1.66 to −0.59), showing moderate-to-large effects. Subgroup analyses showed that mindfulness, music therapy, dance therapy, and Reiki yielded greater psychological benefits than yoga and Qigong. Longer interventions (≥12 weeks) and studies from Asia showed stronger effects, possibly due to differences in intervention type, cultural familiarity, or adherence. Sensitivity analyses confirmed robust results, and no publication bias was detected.

**Conclusion:**

MBTs offer moderate-to-large benefits and represent safe, low-risk strategies for managing menopausal symptoms. Expressive approaches, including Mindfulness, Music therapy, and Dance therapy, may provide added value for emotional regulation and psychological well-being. Yoga and Qigong provide stable benefits for sleep improvement. High-quality trials are needed to inform clinical guidelines.

**Systematic review registration:**

https://www.crd.york.ac.uk/prospero/, identifier CRD420251103844.

## 1 Introduction

Menopause is clinically defined as the permanent cessation of menstruation for ≥12 consecutive months (1). Ovarian aging induces neurochemical alterations, leading to central nervous system (CNS)-related symptoms including vasomotor manifestations, sleep disturbances, anxiety and depression, migraines, and cognitive changes (2). Sleep disturbances represent a hallmark menopausal symptom and global health concern (3), serving as a risk factor for depression and anxiety in menopausal women (4). Depression and anxiety frequently co-occur, with significantly increased risk during perimenopause and menopausal transition (5). These health conditions impair women’s psychosocial functioning (interpersonal relationships, work capacity) and overall quality of life (6, 7). Thus, sleep, depression, and anxiety management are vital during menopause.

Menopause treatment options comprise: Hormone therapy (HT), non-hormonal drugs, and non-drug therapies. HT effectively reduces symptoms but elevates risks of breast cancer, stroke, and thrombosis (8). HT is indicated solely for women aged <60 years with no history of coronary artery disease or breast cancer (9). Thus, when HT risks exceed potential benefits, alternative treatment modalities should be considered. MBTs are considered a form of complementary and alternative medicine and have been widely applied in the treatment of various health conditions (10, 11). MBTs have been defined as focusing on the interactions among the brain, mind, body and behavior. The aim of MBTs is to enhance the capacity for self-knowledge, self-care and to provide tools that can improve coping, mood and quality of life (12).

Recent evidence supports mind–body interventions as effective therapies for menopausal symptoms. However, the findings across studies regarding the effects of different mind–body interventions on menopausal symptoms have been inconsistent (13). Existing meta-analyses have demonstrated the health benefits of MBTs in menopausal women. For example, Xu et al. reported significant improvements in menopausal health outcomes following mind–body exercise interventions. However, these reviews did not include or evaluate art-based therapies, leaving this area underexplored (14). The British Association of Art Therapists (BAAT) defines art therapy as “a form of psychotherapy that uses art media as its primary mode of communication (15). Art therapy, which falls under the category of psychological therapies within MBTs, has been largely overlooked in existing research (16, 17). Shorey et al. found exercise and MBTs effective for menopausal quality of life and depression in Asian women (18), but not hot flashes, with limited generalizability due to ethnic homogeneity. While other researchers have investigated various mind–body exercises (19), sleep assessment was neglected in the study. Sleep has been recognized as a priority outcome in menopause trials based on international, consumer-driven consensus (20).

This study focuses on both physical and psychological dimensions of MBTs. By integrating previous randomized controlled trials (RCTs), this meta-analysis refined evidence on optimal mind–body interventions for sleep, depression, and anxiety in peri-, menopausal, and postmenopausal women. The control group received usual care or no intervention. Our aim is to fill the gap in the literature on art therapy. The findings will inform evidence-based clinical interventions and provide practice guidelines for managing menopausal health.

## 2 Methods

This meta-analysis included randomized controlled trials evaluating the effects of mind–body interventions on sleep, depression, and anxiety in menopausal women. The protocol (CRD420251103844) was registered in PROSPERO and followed PRISMA guidelines.

### 2.1 Search strategy

This meta-analysis was conducted in accordance with the rigorous methodological standards outlined in the Preferred Reporting Items for Systematic Reviews and Meta-Analyses (PRISMA) guidelines.

A comprehensive literature search was conducted in PubMed, the Cochrane Library, Embase, and Web of Science databases, covering studies published in English from database inception to October 10, 2025. The search strategy employed keywords related to “MBTs” and “menopause.” The detailed search strategies are provided in Supplementary Table S1. In addition, the reference lists of all included studies and relevant systematic reviews were manually screened to ensure that no eligible studies were missed.

### 2.2 Inclusion and exclusion criteria

Inclusion criteria were as follows: (i) participants were perimenopausal or postmenopausal women without other underlying medical conditions; (ii) the intervention group received MBTs, such as Exercise therapy, Tai Chi, Yoga, Pilates, Qigong, Baduanjin, Art therapy, Music therapy, Dance, or Mindfulness-based stress reduction, while the control group received only usual care or rehabilitation, or engaged in routine daily activities without any specific intervention; (iii) the study reported at least one of the following outcomes: sleep quality, depression, or anxiety; (iv) the study employed a randomized controlled trial (RCT) design; (v) the article was published in English.

Exclusion criteria were as follows: (i) studies that did not include at least one of the following outcome measures: sleep quality, depression, or anxiety; (ii) studies with incomplete or missing data; (iii) non-RCTs and other types of publications (e.g., case reports, commentaries, and reviews).

### 2.3 Data extraction and quality assessment

Initially, the selected articles were imported into EndNote X9, and duplicate records were independently removed by two reviewers. Subsequently, titles and abstracts were screened based on predefined inclusion and exclusion criteria to identify eligible studies. Two reviewers independently extracted data from the included studies. The extracted data covered the following domains: (1) basic study information, including the first author, year of publication, and country; (2) participant characteristics, including the number and age of participants in both the intervention and control groups; (3) study design details, such as type of intervention, intervention components (duration, frequency, and length of intervention); (4) outcome measures and related data before and after the intervention, including measurement tools and quantitative results; and (5) specific outcomes assessed, including sleep quality (Pittsburgh Sleep Quality Index [PSQI] (21), Insomnia Severity Index [ISI] (22), Women’s Health Initiative Insomnia Rating Scale [WHIIRS]) (23), depression (Depression Anxiety Stress Scale-21 [DASS-21] (24), Hospital Anxiety and Depression Scale [HADS] (25), Beck Depression Inventory [BDI] (26), and other validated measures), anxiety (Depression Anxiety Stress Scale-21 [DASS-21] (24), Hospital Anxiety and Depression Scale [HADS] (25), Beck Anxiety Inventory [BAI] (27), and other validated measures) scores. Means and standard deviations (SDs) were extracted; if unavailable, SDs were derived from standard errors (SEs) or 95% confidence intervals (CIs). When necessary, authors were contacted to obtain missing data. Any discrepancies were resolved through discussion with a third reviewer.

The quality of the included RCTs was assessed using the revised Cochrane Risk of Bias tool for randomized trials (RoB 2) (28). The Cochrane RoB 2 tool was selected because it provides a standardized and widely accepted framework for assessing bias in randomized controlled trials, which comprises five domains. Each domain was rated as “low risk,” “some concerns,” or “high risk” of bias. Two reviewers (A and B) independently conducted the risk of bias assessments. Any discrepancies between the reviewers were resolved through discussion with a third reviewer.

### 2.4 Statistical analysis

This study employed Review Manager version 5.3.0 and Stata version 17 to conduct the meta-analysis.

All outcome data were continuous variables. According to the Cochrane Handbook, a fixed-effects model was used when heterogeneity was low or absent (I^2^ ≤ 50%, *p* ≥ 0.1) (29), while a random-effects model was applied in cases of substantial heterogeneity (I^2^ > 50%, *p* < 0.1). In this study, a random-effects model was employed to calculate the SMD and the corresponding 95% confidence interval (CI) for each outcome, as different measurement scales were used across studies. Given the variability in study designs and outcome measurement scales, a random-effects model was selected as the primary analytical approach to account for between-study heterogeneity. The SMD was interpreted as a small (≈0.2), medium (≈0.5), or large (≈0.8) effect size, following established thresholds (30). A negative SMD indicated a favorable effect of the intervention. Statistical significance was determined at *p* ≤ 0.05. Between-study heterogeneity was assessed using the *p*-value and I^2^ statistic.

When the effect size was ≥10, publication bias was assessed through funnel plots and Egger’s test (31). Sensitivity analyses were conducted by sequentially removing individual studies to evaluate the consistency of the pooled effect size and the stability of the findings.

Subgroup analyses will be conducted for sleep, depression, and anxiety outcomes. Potential sources of heterogeneity will be explored across four dimensions: intervention category, intervention duration (<12 weeks vs. ≥12 weeks), region (Asian vs. non-Asian), and study quality (low risk vs. some concerns). To explore category-specific effects, we will further refine the subgroup analyses and present results stratified by specific intervention types, which will provide more meaningful insights.

## 3 Results

### 3.1 Literature search

A total of 11,693 records were identified from database searches (PubMed, Cochrane Library, Embase, Web of Science; *n* = 11,675) and other sources (*n* = 18), then imported into EndNote X9 for reference management. After removing 3,082 duplicates, 8,611 articles were excluded based on title and abstract screening. These exclusions included 93 non-RCT studies, 7,572 articles unrelated to the target disease population, 377 studies with incompatible intervention methods, 167 studies lacking relevant outcome measures, 3 dissertations, and 287 review articles.

A total of 112 full-text articles were assessed for eligibility. Of these, 94 were excluded for the following reasons: not randomized controlled trials (*n* = 18), incompatible interventions (*n* = 2), ineligible study populations (*n* = 9), absence of relevant outcome measures (*n* = 43), lack of a blank control group (*n* = 20), and conference abstracts (*n* = 2).

**Figure 1:**
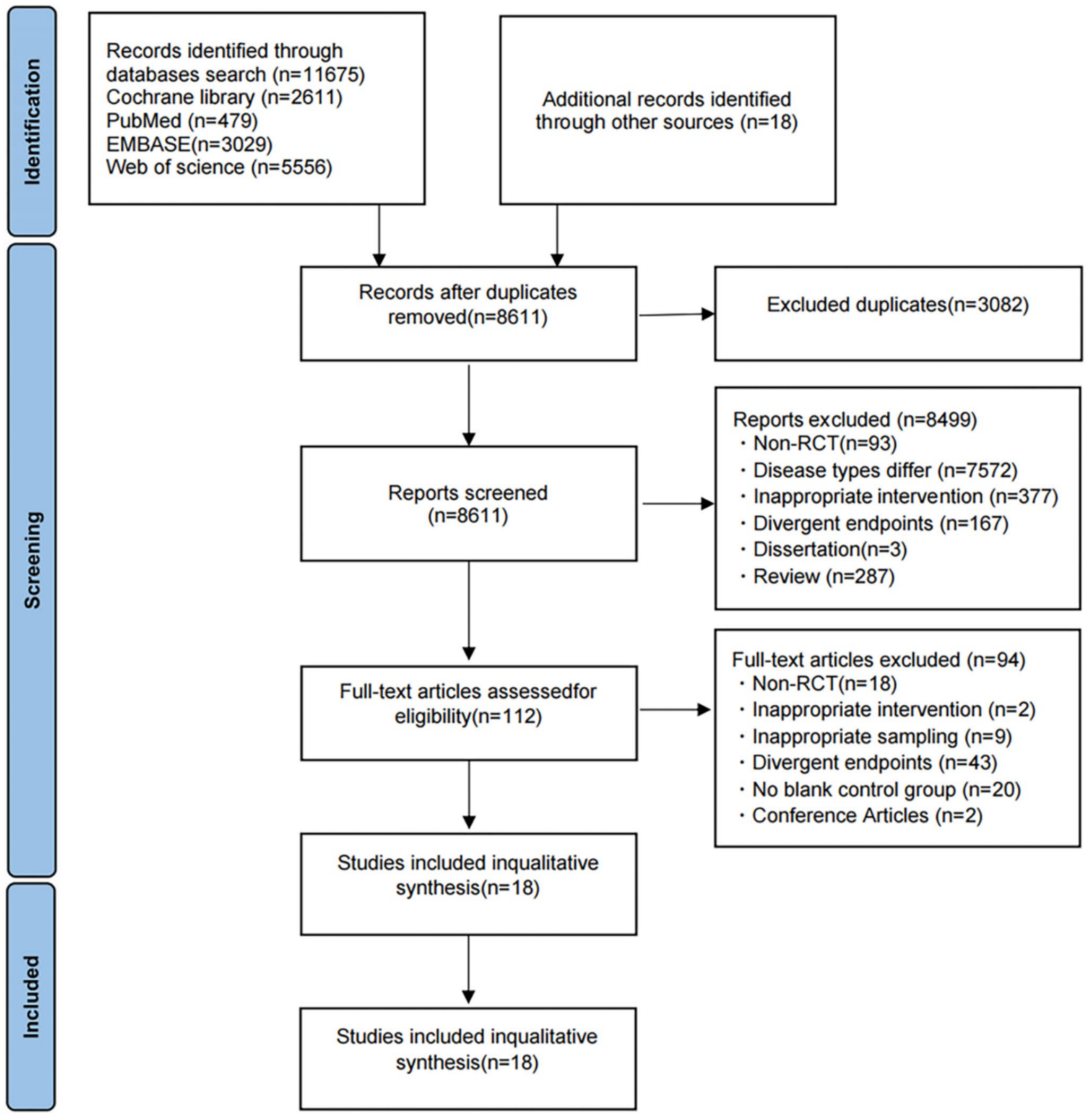
The PRISMA flowchart for included RCTs.

Finally, 18 studies met the inclusion criteria and were included in the present systematic review and meta-analysis. The study selection process, along with reasons for exclusion at each stage, is presented in Figure 1 (PRISMA flow diagram).

### 3.2 Study characteristics

A total of 18 RCTs were included in this study. The MBTs implemented in the experimental groups comprised 5 mindfulness-based interventions (32–36), 6 yoga interventions (37–42), 2 Pilates interventions (43, 44), 2 music therapy interventions (45, 46), 1 Qigong intervention (47), 1 dance intervention (48), 1 Reiki intervention (49). The included studies involved 1,570 participants from 9 different countries. Among the 18 included trials, not all reported all three outcomes; therefore, the number of studies included in each meta-analysis differed by outcome (sleep: 13; depression: 12; anxiety: 9).

The duration of interventions ranged from 4 to 24 weeks, with a frequency of 2 to 8 sessions per week, and each session lasting between 15 and 60 min. Mindfulness programs included a 7-h intensive weekend practice and weekly mindfulness sessions totaling 150 min. The yoga interventions typically involved 90 min of practice per week. Participants in the control groups engaged only in routine daily activities without any specific exercise intervention.

Primary outcomes assessed included sleep quality, depression, and anxiety, measured using a variety of validated assessment tools. Table 1 presents the detailed characteristics of the included studies.

**Table 1:** Characteristics of the 18 randomized controlled studies.

Included literature	Country	Population	Experimental group			Control group			Outcome measures
			Sample size	Age mean (SD)	Exercise intervention	Sample size	Age mean (SD)	Exercise intervention	
Amin et al. 2025 (32)	Egypt	Menopausal women	60	40–60	MBIS: 4 weeks, (7 sessions, 90 min/session, 2×/week)	60	40–60	Individual counseling 30-min (incl. Health education)	b1, c1
Wong et al. 2018 (36)	China	Perimenopausal and postmenopausal women	69	51.9 ± 3.0	MBSR: 8 weeks program (2.5 h/week) + 40 min/day home practice	70	52.1 ± 3.2	MEC: 8-week education & stretching (2.5 h/week) + 40 min/day home practice	b2, c2
Gordon et al. 2021 (35)	Canada	Early Perimenopause women	52	48.7 ± 3.7	MBSR: 8 weeks, (150 min/week + retreat; mindfulness, yoga, group work)	52	48.7 ± 3.0	No Intervention	a1, b3, c3
Darehzereshki et al. 2022 (34)	Iran	Postmenopausal women	28	52.9 ± 4.4	MBSR: 8 weeks, 1×/week, 120 min/session + homework	28	53.6 ± 4.1	Menopausal health education: 8 weeks 1×/week, 120 min/session	a1
Carmody et al. 2011 (33)	American	Perimenopausal and early postmenopausal women	57	52.5 ± 5.4	MBSR: 8 weeks 1×/week, 2.5 h + 1 all-day retreat; 45 min/day home practice (6 days/week)	48	53.8 ± 4.4	No Intervention	a3, c4
Lu et al. 2020 (37)	China	Postmenopausal women	52	50.56 ± 3.27	Yoga + info support: 24 weeks (3×/week, 60 min)	54	50.74 ± 2.95	Usual care; no other exercise for 6 months	a1, b5, c5
Susanti et al. 2022 (38)	Indonesia	Perimenopausal and postmenopausal women	95	52.39 ± 4.23	Yoga: 20 weeks 3×/week, 75 min/session; home-based with guidance	92	52.57 ± 3.90	No Intervention	a1
Buchanan et al. 2022 (39)	American	Late transition and postmenopausal women	52	55.3 ± 3.9	Yoga: 12 weeks 1×/week, 90 min + 20 min/day home practice (poses & Yoga Nidra)	80	54.2 ± 3.7	No Intervention	a1
Afonso et al. 2012 (40)	Brazil	Postmenopausal women	15	50–65	Yoga: 16 weeks 2×/week, 60 min/session; includes asanas, bhastrika, and relaxation	15	50–65	No intervention; monthly check-in via phone	a2, b6, c6
Aksoy-Can et al. 2025 (42)	Turkey	Natural menopause period	18	51.39 ± 3.65	Yoga: 2 sessions/week for 4 weeks; 40–45 min/session (4 steps)	18	52.72 ± 4.39	No intervention	a1
Portella et al. 2021 (41)	Brazil	Perimenopausal women	18	46.7 ± 4.3	Meditation (Raja Yoga): 8 weeks 45 min/day + sleep hygiene	15	48.6 ± 5.4	Sleep hygiene only (no meditation)	a1, b7
Aibar-Almazán et al. 2019 (44)	Spain	Postmenopausal women	55	69.98 ± 7.83	Pilates: 12 weeks, 2×/week, 60 min/session; (progressive intensity; supervised)	52	66.79 ± 10.14	Usual lifestyle + physical activity guidelines; no exercise; phone follow-up	a1, b4, c4
Ahmadinezhad et al. 2017 (43)	Iran	Postmenopausal women	36	51.08 ± 2.61	Pilates: 6 weeks, 3×/week, 60 min/session	36	50.72 ± 2.51	No intervention	a1
Ugurlu et al. 2024 (46)	Turkey	Menopausal women	30	53.4 ± 4.75	Music therapy: 5 weeks 2×/day (15 min AM + 15 min PM)	31	53.4 ± 4.75	No intervention	a1, b6
Koçak et al. 2022 (45)	Turkey	Postmenopausal women	21	59.1 ± 4.2	Music therapy: 6 weeks 1×/day, 15 min (Büzürk mode)	27	56.5 ± 6.5	No intervention	b6
Martins et al. 2025 (48)	Brazil	Menopausal women	15	40–59	Jazz Dance: 16 weeks, 2×/week, 60 min; (BPM-based intensity; Borg Scale)	20	40–59	Usual care + monitoring (no dance)	b4, c4
Carcelén-Fraile et al. 2022 (47)	Spain	Postmenopausal women	57	69.70 ± 6.15	Baduanjin Qigong: 12 weeks, 2×/week, 60 min/session (warm-up, Qigong, cooldown)	60	69.75 ± 6.76	Usual lifestyle + physical activity recommendations (no exercise program)	a1, b4, c4
Sabancı Baransel et al. 2025 (49)	Turkey	Postmenopausal women	41	49.36 ± 3.03	Reiki:1 session/week for 4 weeks (total 4 sessions)	41	49.97 ± 3.16	Usual care	b6

a1: PSQI, Pittsburgh Sleep Quality Index; a2: ISI, Insomnia Severity Index; a3: WHIIRS, Women’s Health Initiative Insomnia Rating Scale; b1: DASS-21, Depression Anxiety Stress Scale 21 - Depression Subscale; b2: GCS-Depression, Modified Greene Climacteric Scale - Depression Subscale; b3: CES-D, Center for Epidemiologic Studies Depression Scale; b4: HADS, Hospital Anxiety and Depression Scale Depression Scale - Depression Subscale; b5: SDS, Self-rating Depression Scale; b6: BDI, Beck Depression Inventory; b7: KMI, Kupperman Menopausal Index - Depression; c1: DASS-21, Depression Anxiety Stress Scale 21 - Anxiety Subscale; c2: GCS-Anxiety, Modified Greene Climacteric Scale - Anxiety Subscale; c3: STAI-T, Spielberger Trait Anxiety Inventory; c4: HADS, Hospital Anxiety and Depression Scale - Anxiety Subscale; c5: SAS, Self-rating Anxiety Scale; c6: BAI, Beck Anxiety Inventory.

### 3.3 Quality assessment

**Figure 2:**
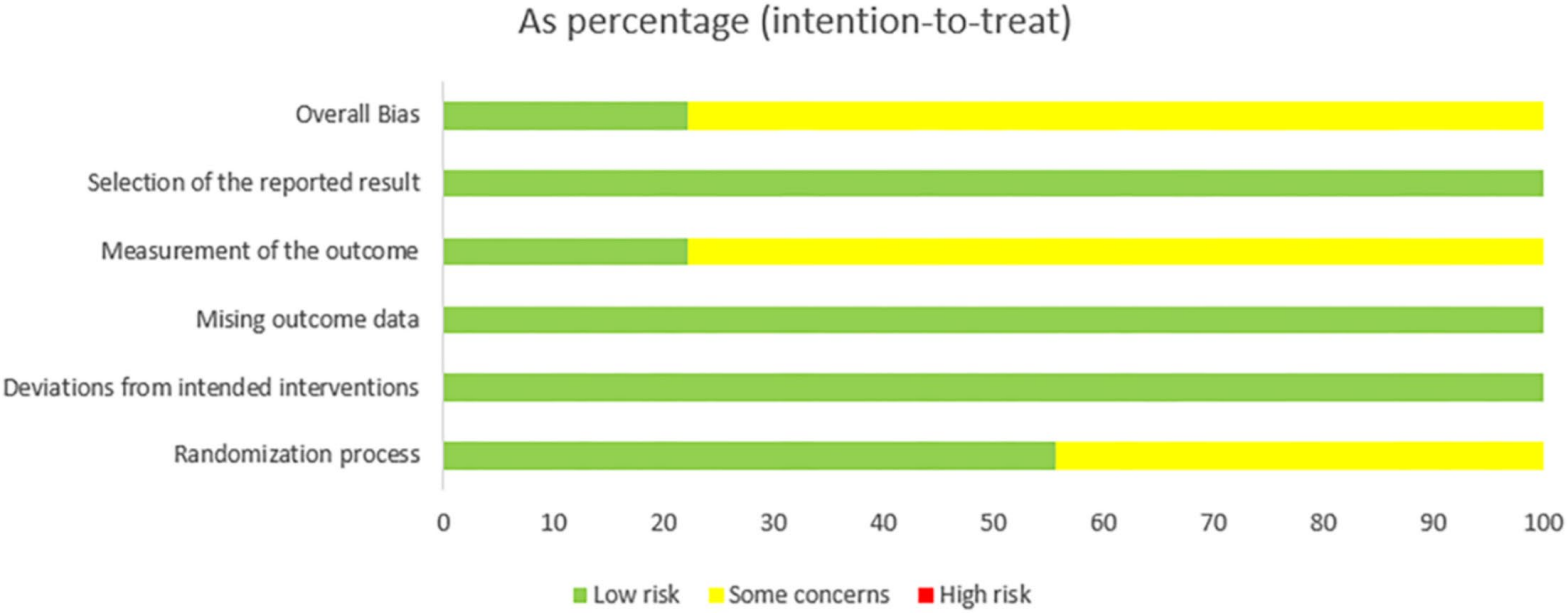
Risk of bias summary.

**Figure 3:**
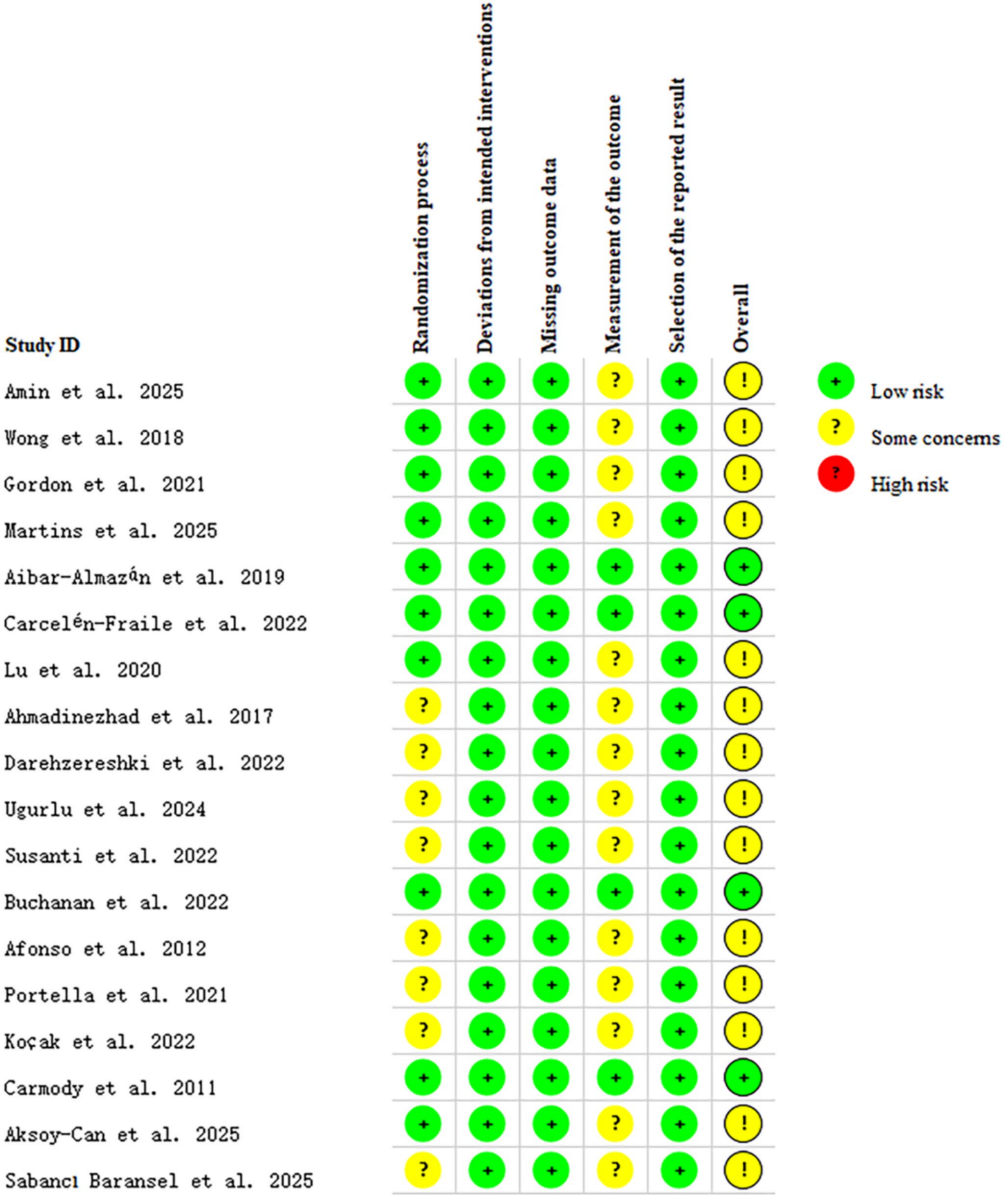
Graph depicting the risk of bias.

Among the 18 included studies, 14 were rated as having “some concerns,” and 4 were rated as “low risk” overall. Regarding the randomization process, 10 studies were assessed as “low risk” and 8 as “some concerns.” For deviations from intended interventions and missing outcome data, all 18 studies were rated as “low risk.” In the domain of measurement of the outcome, 4 studies were judged to be “low risk” and 14 as “some concerns.” Inter-rater agreement between the two reviewers was considered high. Since all studies were prospectively registered, the selection of the reported result was judged to be at “low risk” of bias across all included studies (see Figures 2, 3).

### 3.4 Meta-analysis of the outcome measures

**Figure 4:**
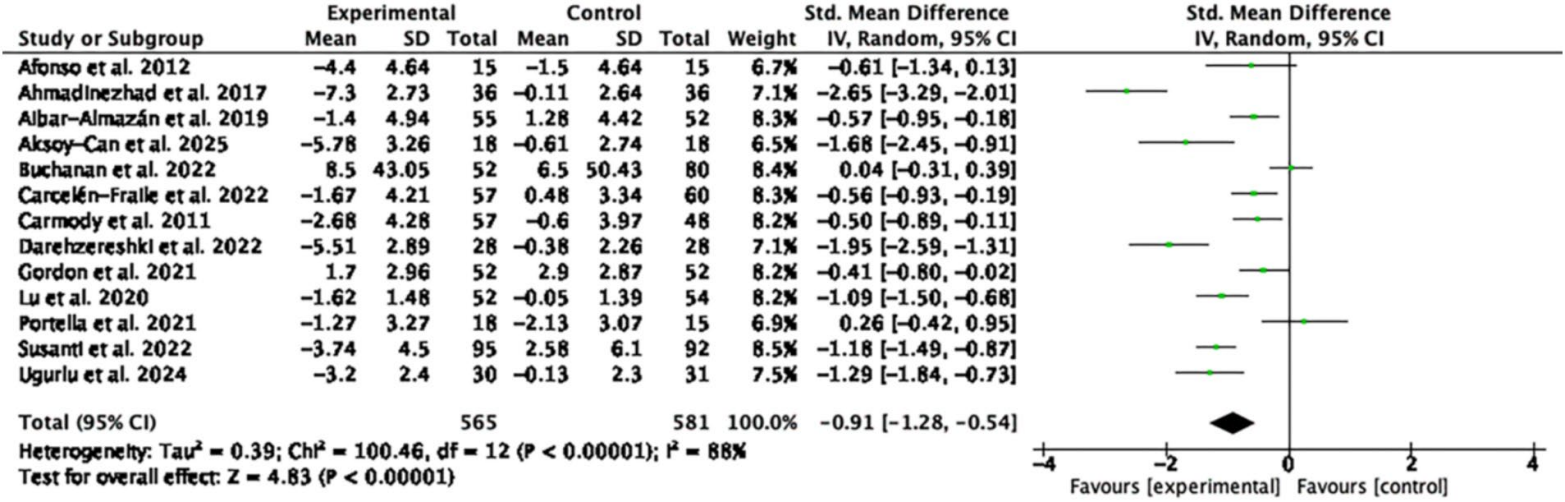
Forest plot of the effects of MBTs on sleep quality.

A total of 13 RCTs involving 1,146 participants were included in the meta-analysis of sleep outcomes. The pooled SMD was −0.91 (95% CI: −1.28 to −0.54; *p* < 0.00001; I^2^ = 88%), indicating a statistically significant moderate-to-large effect in favor of MBTs compared to the control group (Figure 4).

**Figure 5:**
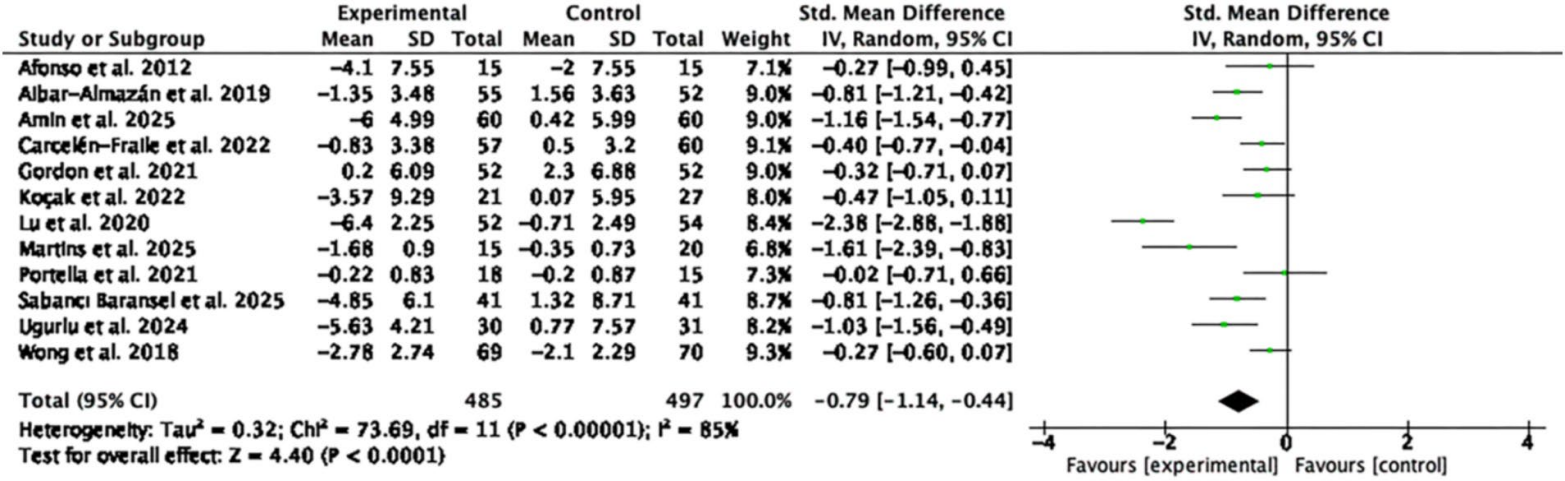
Forest plot of the effects of MBTs on depression.

A total of 12 RCTs involving 982 participants were included in the meta-analysis of depression outcomes. The pooled SMD was −0.79 (95% CI: −1.14 to −0.44; *p* < 0.0001; I^2^ = 85%), indicating a statistically significant moderate effect in favor of MBTs compared to the control group (Figure 5).

**Figure 6:**
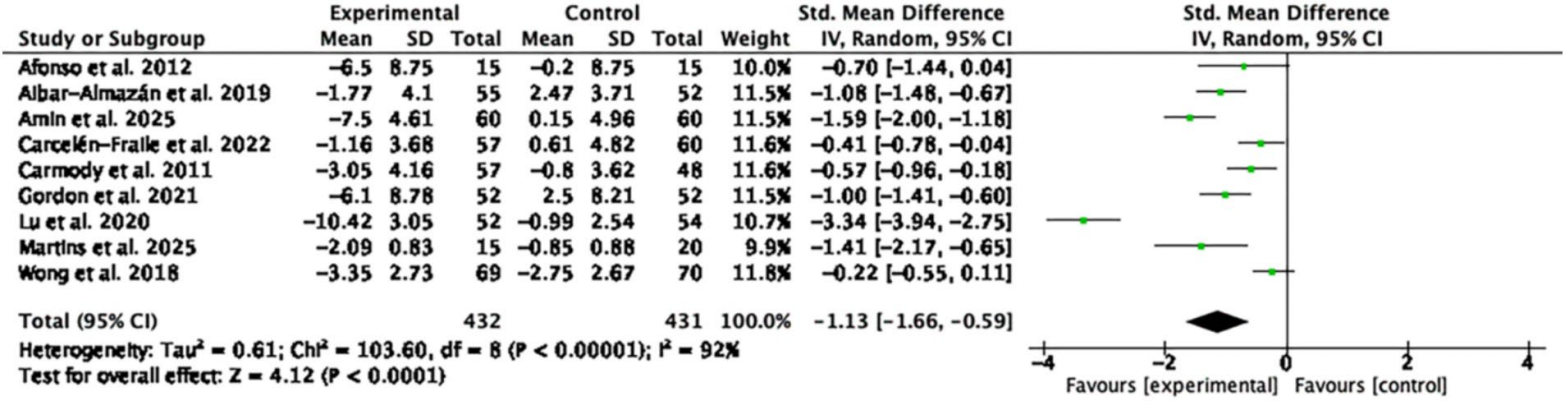
Forest plot of the effects of MBTs on anxiety.

A total of 9 RCTs involving 863 participants were included in the meta-analysis of anxiety outcomes. The pooled SMD was −1.13 (95% CI: −1.66 to −0.59; *p* < 0.0001; I^2^ = 92%), indicating a statistically significant effect in favor of MBTs compared to the control group (Figure 6).

Substantial heterogeneity was observed across sleep, depression, and anxiety outcomes, which may be attributable to differences in intervention type, duration, and outcome assessment tools.

### 3.5 Subgroup analysis

To further explore potential sources of heterogeneity, subgroup analyses were conducted based on four factors: intervention category, intervention duration, region, and study quality. The results are presented in Tables 2–4.

**Table 2:** Subgroup analyses of sleep quality between MBTs and control groups.

Categorical moderator	Level	No. of studies	SMD	95% CI	Overall effect (P)	Subgroup differences (P)	Between-group Heterogeneity	
							I^2^, %	*p*-value
Study design moderators								
Intervention category	Yoga	6	−0.70	−1.28, −0.13	P = 0.02	P = 0.26	89	*p*<0.00001
	Mindfulness	3	−0.91	−1.69, −0.12	P = 0.02		89	P = 0.0001
	Pilates	2	−1.59	−3.63, −0.45	P = 0.13		97	*p*<0.00001
	Qigong	1	−0.56	−0.93, −0.19	P = 0.003		/	/
	Music	1	−1.29	−1.84, −0.73	*p*<0.00001		/	/
Study quality	Low	4	−0.39	−0.69, −0.09	P = 0.01	P = 0.007	62	*p* = 0.05
	Some concerns	9	−1.17	−1.65, −0.69	*p*<0.00001		87	*p*<0.00001
Intervention duration	<12 weeks	6	−1.29	−2.10, −0.49	P = 0.002	P = 0.14	91	*p*<0.00001
	≥12 weeks	7	−0.63	−0.97, −0.28	P = 0.004		81	*p*<0.0001
Region	Asia	6	−1.59	−2.03, −1.15	*p*<0.00001	*p*<0.00001	78	P = 0.0005
	Non-Asia	7	−0.35	−0.58, −0.13	P = 0.002		48	P = 0.07

**Table 3:** Subgroup analysis of depression between MBTs and control groups.

Categorical moderator	Level	No. of studies	SMD	95% CI	Overall effect (P)	Subgroup differences (P)	Between-group heterogeneity	
							I^2^, %	*p*-value
Study design moderators								
Intervention category	Yoga	3	−0.91	−2.51, −0.70	P = 0.27	*p* = 0.20	95	*p*<0.00001
	Mindfulness	3	−0.58	−1.13, −0.02	P = 0.04		85	P = 0.001
	Music	2	−0.76	−1.30, −0.22	P = 0.006		47	*p* = 0.17
	Pilates	1	−0.81	−1.21, −0.42	P<0.0001		/	/
	Qigong	1	−0.40	−0.77, −0.04	P = 0.03		/	/
	Dance	1	−1.61	−2.39, −0.83	P<0.0001		/	/
	Reiki	1	−0.81	−1.26, −0.36	P = 0.0004		/	/
Study quality	Low	2	−0.60	−1.00, −0.20	P = 0.003	P = 0.44	55	P = 0.13
	Some concerns	10	−0.83	−1.27, −0.40	P = 0.0002		87	*p*<0.00001
Intervention duration	<12 weeks	6	−0.65	−1.01, −0.29	P = 0.0004	P = 0.42	72	P = 0.004
	≥12 weeks	6	−0.95	−1.60, −0.31	P = 0.004		91	*p*<0.00001
Region	Asia	5	−0.99	−1.72, −0.25	P = 0.008	P = 0.42	92	*p*<0.00001
	Non-Asia	7	−0.65	−1.00, −0.30	P = 0.003		73	P = 0.001

**Table 4:** Subgroup analysis of anxiety between MBTs and control groups.

Categorical moderator	Level	No. of studies	SMD	95% CI	Overall effect (P)	Subgroup differences (P)	Between-group heterogeneity	
							I^2^, %	*p*-value
Study design moderators								
Intervention category	Yoga	2	−2.03	−4.62, −0.56	P = 0.12	P = 0.05	97	*p*<0.00001
	Mindfulness	4	−0.84	−1.42, −0.25	P = 0.005		89	*p*<0.00001
	Pilates	1	−1.08	−1.48, −0.67	P<0.00001		/	/
	Qigong	1	−0.41	−0.78, −0.04	P = 0.03		/	/
	Dance	1	−1.41	−2.17, −0.65	P = 0.0003		/	/
Study quality	Low	3	−0.68	−1.07, −0.29	P = 0.0006	P = 0.14	67	P = 0.05
	Some concerns	6	−1.37	−2.21, −0.53	P = 0.001		94	*p*<0.00001
Intervention duration	<12 weeks	3	−0.79	−1.58, −0.00	P = 0.05	P = 0.35	92	*p*<0.00001
	≥12 weeks	6	−1.31	−2.08, −0.55	P = 0.0008		93	*p*<0.00001
Region	Asia	2	−1.77	−4.83, −1.29	P = 0.26	P = 0.60	99	*p*<0.00001
	Non-Asia	7	−0.95	−1.29, −0.61	P<0.00001		74	*p* = 0.0007

The Table 2 presents a subgroup analysis of the effects of mind–body interventions on sleep quality compared to control groups, considering moderators such as intervention category, intervention duration, study quality and region.

Subgroup analyses by intervention category indicated significant improvements in sleep quality for Yoga (SMD = −0.70, 95% CI: −1.28 to −0.13, *p* = 0.02), Mindfulness (SMD = −0.91, 95% CI: −1.69 to −0.12, *p* = 0.02), and Music therapy (SMD = −1.29, 95% CI: −1.84 to −0.73, *p* < 0.00001). Pilates (SMD = −1.59, 95% CI: −3.63 to 0.45, *p* = 0.13) and Qigong (SMD = −0.56, 95% CI: −0.93 to −0.19, *p* = 0.003) also showed beneficial trends, though the effect for Pilates was not statistically significant. Studies with “some concerns” in methodological quality demonstrated greater effects (SMD = −1.17, 95% CI: −1.65 to −0.69, *p* < 0.00001) compared to low-quality studies (SMD = −0.39, 95% CI: −0.69 to −0.09, *p* = 0.01). Regarding intervention duration, interventions lasting less than 12 weeks yielded a stronger improvement in sleep quality (SMD = −1.29, 95% CI: −2.10 to −0.49, *p* = 0.002) than those lasting 12 weeks or longer (SMD = −0.63, 95% CI: −0.97 to −0.28, *p* = 0.004), although the between-group difference was not statistically significant (*p* = 0.14). By region, studies conducted in Asia showed markedly stronger effects (SMD = −1.59, 95% CI: −2.03 to −1.15, *p* < 0.0001) compared to those in non-Asian regions (SMD = −0.35, 95% CI: −0.58 to −0.13, *p* = 0.002), with statistically significant between-group differences (*p* < 0.0001).

While MBTs significantly improve sleep quality, effect sizes vary by intervention category, study quality, intervention duration, and geographic region—with the most pronounced moderator effect observed for region.

Table 3 presents a subgroup analysis of the effects of MBTs on depression, stratified by study design moderators including intervention category, intervention duration, study quality and region.

Subgroup analyses by intervention category showed significant reductions in depression for Mindfulness (SMD = −0.58, 95% CI: −1.13 to −0.02, *p* = 0.04), Music therapy (SMD = −0.76, 95% CI: −1.30 to −0.22, *p* = 0.006), Pilates (SMD = −0.81, 95% CI: −1.21 to −0.42, *p* < 0.0001), Qigong (SMD = −0.40, 95% CI: −0.77 to −0.04, *p* = 0.03), Dance therapy (SMD = −1.61, 95% CI: −2.39 to −0.83, *p* < 0.0001), and Reiki (SMD = −0.81, 95% CI: −1.26 to −0.36, *p* = 0.0004). Although Yoga also showed a favorable trend (SMD = −0.91, 95% CI: −2.51 to −0.70), the result did not reach statistical significance (*p* = 0.27). Studies with “some concerns” in risk of bias had greater effects (SMD = −0.83, 95% CI: −1.27 to −0.40, *p* = 0.0002) compared to low-quality studies (SMD = −0.60, 95% CI: −1.00 to −0.20, *p* = 0.003). Regarding exercise duration, interventions lasting less than 12 weeks produced moderate improvements in depression (SMD = −0.65, 95% CI: −1.01 to −0.29, *p* = 0.0004), while those lasting 12 weeks or longer showed slightly larger effects (SMD = −0.95, 95% CI: −1.60 to −0.31, *p* = 0.004), with no significant subgroup difference (*p* = 0.42). Regionally, Asian studies demonstrated stronger effects (SMD = −0.99, 95% CI: −1.72 to −0.25, *p* = 0.008) compared to non-Asian studies (SMD = −0.65, 95% CI: −1.00 to −0.30, *p* = 0.003); however, no significant subgroup differences were detected across categories (*p* > 0.05).

MBTs demonstrated beneficial effects in reducing depression across various study characteristics. While no statistically significant subgroup differences were found, variability in intervention category, study quality, intervention duration, and geographic region may contribute to the observed heterogeneity.

Table 4 presents a subgroup analysis examining the effects of MBTs on anxiety across various study design moderators, including intervention category, intervention duration, study quality and region.

Subgroup analyses by intervention category showed significant reductions in anxiety for Mindfulness (SMD = −0.84, 95% CI: −1.42 to −0.25, *p* = 0.005), Pilates (SMD = −1.08, 95% CI: −1.48 to −0.67, *p* < 0.00001), Qigong (SMD = −0.41, 95% CI: −0.78 to −0.04, *p* = 0.03), and Dance therapy (SMD = −1.41, 95% CI: −2.17 to −0.65, *p* = 0.0003). Although Yoga also demonstrated a large effect size (SMD = −2.03, 95% CI: −4.62 to −0.56), this result was not statistically significant (*p* = 0.12), likely due to limited sample size. Studies rated with “some concerns” in quality yielded stronger effects (SMD = −1.37, 95% CI: −2.21 to −0.53, *p* = 0.001) compared to low-quality studies (SMD = −0.68, 95% CI: −1.07 to −0.29, *p* = 0.0006). Longer interventions (≥12 weeks) produced greater reductions in anxiety (SMD = −1.31, 95% CI: −2.08 to −0.55, *p* = 0.0008) than shorter interventions. Non-Asian studies showed significant improvements (SMD = −0.95, 95% CI: −1.29 to −0.61, *p* < 0.00001), while Asian studies reported a non-significant effect (SMD = −1.77, 95% CI: −4.83 to 1.29, *p* = 0.26).

In conclusion, MBTs were generally effective in reducing anxiety regardless of the intervention category, study quality, intervention duration, or geographic region. Although no statistically significant subgroup differences were detected, substantial heterogeneity indicates that these factors may still influence effect sizes.

Table 5 presents a subgroup analysis examining the comparative effects of different MBTs on sleep, depression, and anxiety outcomes.

**Table 5:** Subgroup analysis of the comparative effects of different MBTs on sleep, depression, and anxiety.

Outcome measures	Intervention category	No. of studies	SMD	95% CI	Overall effect (P)	Subgroup differences (P)	Between-group heterogeneity	
							I^2^, %	*p*-value
Sleep	Yoga	6	−0.70	−1.28, −0.13	P = 0.02	P = 0.26	89	*p*<0.00001
	Mindfulness	3	−0.91	−1.69, −0.12	P = 0.02		89	P = 0.0001
	Pilates	2	−1.59	−3.63, −0.45	P = 0.13		97	*p*<0.00001
	Qigong	1	−0.56	−0.93, −0.19	P = 0.003		/	/
	Music	1	−1.29	−1.84, −0.73	*p*<0.00001		/	/
Depression	Yoga	3	−0.91	−2.51, −0.70	P = 0.27	P = 0.20	95	*p*<0.00001
	Mindfulness	3	−0.58	−1.13, −0.02	P = 0.04		85	P = 0.001
	Music	2	−0.76	−1.30, −0.22	P = 0.006		47	P = 0.17
	Pilates	1	−0.81	−1.21, −0.42	*p*<0.0001		/	/
	Qigong	1	−0.40	−0.77, −0.04	P = 0.03		/	/
	Dance	1	−1.61	−2.39, −0.83	*p*<0.0001		/	/
	Reiki	1	−0.81	−1.26, −0.36	P = 0.0004		/	/
Anxiety	Yoga	2	−2.03	−4.62, −0.56	P = 0.12	P = 0.05	97	*p*<0.00001
	Mindfulness	4	−0.84	−1.42, −0.25	P = 0.005		89	*p*<0.00001
	Pilates	1	−1.08	−1.48, −0.67	P<0.00001		/	/
	Qigong	1	−0.41	−0.78, −0.04	P = 0.03		/	/
	Dance	1	−1.41	−2.17, −0.65	P = 0.0003		/	/

For sleep outcomes, all interventions demonstrated beneficial effects, with Music therapy (SMD = −1.29, 95% CI: −1.84 to −0.73, *p* < 0.0001) and Pilates (SMD = −1.59, 95% CI: −3.63 to −0.45) showing the largest effect sizes, followed by Mindfulness and Yoga. Qigong also yielded a significant but smaller improvement (SMD = −0.56, *p* = 0.003).

For depression, all included MBTs showed moderate-to-large benefits. Dance therapy (SMD = −1.61, 95% CI: −2.39 to −0.83), Music therapy (SMD = −0.76, *p* = 0.006), and Reiki (SMD = −0.81, *p* = 0.0004) exhibited stronger effects than exercise-based interventions such as Qigong and Yoga.

For anxiety, Pilates (SMD = −1.08, *p* < 0.0001) and Dance therapy (SMD = −1.41, *p* = 0.0003) produced the largest reductions, followed by Mindfulness and Qigong.

Music therapy, Dance therapy, Mindfulness appeared to yield stronger psychological benefits than Yoga and Qigong. Between-group heterogeneity was high across most outcomes (I^2^ > 85%), reflecting variations in modality characteristics and study designs.

### 3.6 Publication bias

**Figure 7:**
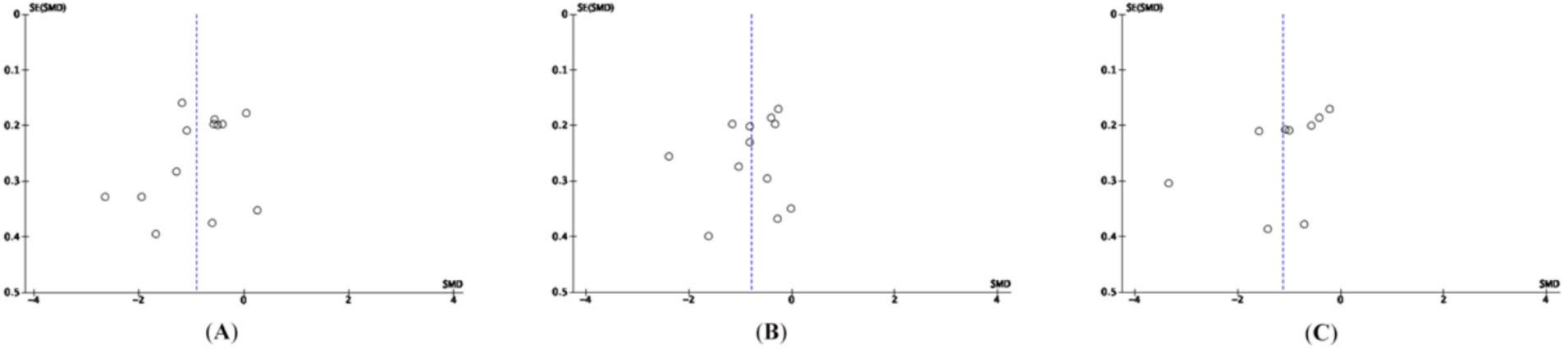
Bias analysis **(A)** sleep **(B)** depression **(C)** anxiety.

Publication bias related to sleep quality, depression, and anxiety was assessed using a combination of subjective funnel plot inspection and the objective Egger’s regression test. As shown in Figure 7, the funnel plots were visually examined for symmetry to identify potential publication bias. The distribution of studies appeared to be approximately symmetrical on both sides of the funnel plots. Additionally, Egger’s test did not detect significant publication bias for sleep quality (t = −1.37, 95% CI: −9.92 to 2.30, *p* = 0.197), depression (t = −0.75, 95% CI: −9.09 to 4.51, *p* = 0.471), or anxiety (t = −1.73, 95% CI: −17.67 to 2.75, *p* = 0.128). These findings suggest that there was no substantial evidence of publication bias in the included studies.

### 3.7 Sensitivity analysis

**Figure 8:**
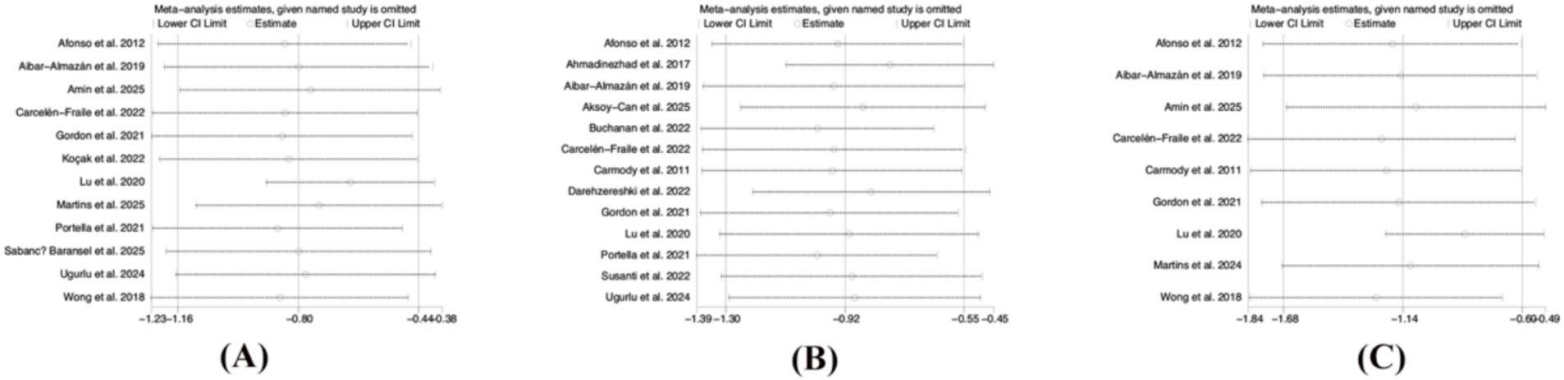
Sensitivity analysis **(A)** sleep **(B)** depression **(C)** anxiety.

As shown in Figure 7, the effect sizes for sleep, depression, and anxiety outcomes did not change substantially after sequential exclusion of individual studies, affirming the stability and credibility of the meta-analysis results in this study (Figure 8).

## 4 Discussion

This systematic review and meta-analysis synthesized evidence from 18 randomized controlled trials (RCTs) involving 1,572 perimenopausal and postmenopausal women, evaluating the efficacy of diverse MBTs such as Yoga, Mindfulness-based stress reduction (MBSR), Qigong, Music therapy, Dance therapy, and Reiki, on sleep disturbances, depression, and anxiety. MBTs demonstrated moderate-to-large beneficial effects across all outcomes, with heterogeneity partially explained by intervention category, intervention duration, study quality and region. Subgroup analyses revealed that several specific modalities—including Mindfulness, Pilates, Music, Dance, and Reiki—were particularly effective in improving depression and anxiety, while Yoga and Qigong showed moderate but consistent benefits for sleep quality. Interventions with longer durations (≥12 weeks) and studies conducted in Asian populations tended to yield greater improvements, possibly reflecting differences in intervention intensity, delivery format, and cultural familiarity. However, other factors—such as regional publication bias, population characteristics, or variations in adherence—may also contribute to these findings.

The substantial heterogeneity observed across outcomes highlights that MBTs should not be regarded as a single, uniform therapeutic category. Instead, their effectiveness appears to depend on the specific modality and the symptom targeted. For instance, music therapy and Pilates were most effective for improving sleep, while dance therapy and Reiki yielded stronger effects for depression. Mindfulness-based interventions consistently reduced both depression and anxiety symptoms, suggesting broader psychological benefits. These findings imply that clinicians should tailor the selection of MBT modalities based on the predominant symptom profile—such as using movement-based interventions (e.g., yoga, Pilates) for somatic complaints and expressive or meditative approaches (e.g., music, dance, mindfulness) for emotional and cognitive disturbances. Future trials should further explore these differential effects through head-to-head comparisons of MBT subtypes and standardized intervention protocols to guide more precise clinical decision-making.

### 4.1 Sleep disturbances

The pooled analysis showed a moderate-to-large improvement in sleep quality among women receiving MBTs compared to controls. These findings align with earlier meta-analyses investigating yoga, Tai Chi, and mindfulness for menopausal sleep problems. For instance, Xu et al. (14) and Cramer et al. (50) demonstrated moderate improvements in sleep quality among midlife women, although their effect sizes were slightly smaller than those observed in the current study. Unlike those reviews, the present meta-analysis incorporates both exercise-based and expressive MBTs (e.g., music, art, and Reiki), thus expanding the scope of interventions evaluated. This broader inclusion may partly explain the stronger overall effect observed here.

The improvement in sleep quality may be linked to the physiological effects of MBTs. These interventions can regulate the hypothalamic pituitary adrenal (HPA) axis, lower cortisol levels, and restore circadian balance (51). MBTs also enhance parasympathetic activity and reduce sympathetic arousal, promoting relaxation and reducing hyperarousal—key factors in sleep regulation (52). MBTs can reduce physiological hyperarousal and promote parasympathetic activity. They also help regulate melatonin and cortisol levels, contributing to better sleep architecture (53, 54).

Among individual MBTs modalities, Yoga, Mindfulness, and Music therapy demonstrated significant improvements in sleep quality, while Pilates and Qigong also showed favorable trends. These findings suggest that both physically oriented (e.g., Yoga, Pilates, Qigong) and meditative or expressive approaches (e.g., Mindfulness, Music therapy) can effectively enhance sleep regulation through different mechanisms. Interventions lasting 12 weeks or longer appeared more effective, supporting the importance of sustained behavioral engagement, as long-term practice may induce neuroplastic adaptations critical for sleep homeostasis (55). Studies conducted in Asian populations reported stronger sleep-related effects, which may be related to cultural familiarity with MBTs, higher adherence, or other regional factors such as publication bias or participant characteristics (18).

Clinically, MBTs can be recommended as non-pharmacologic options to manage sleep disturbances during menopause. Policymakers should consider integrating MBTs into public health initiatives promoting healthy aging (56). Music therapy appears particularly promising, as it enhances parasympathetic activity and reduces sleep-onset latency through rhythmic auditory stimulation and relaxation mechanisms (46). Yoga and Mindfulness-based interventions may improve sleep quality by modulating autonomic balance, reducing stress arousal, and enhancing emotional regulation, while Qigong and Pilates contribute to relaxation and improved sleep efficiency through gentle physical engagement and breathing control. Dance therapy may promote emotional decompression, improve circadian rhythm stability, and increase physical fatigue, all of which facilitate deeper sleep (57). These modalities are especially promising given their adaptability to group or home-based formats, which may increase accessibility and adherence in menopausal populations. Future research should focus on high-quality RCTs with long-term follow-up, objective sleep assessments, and exploration of biological mediators such as stress, inflammation, and hormonal changes (58).

### 4.2 Depression

MBTs were associated with significant reductions in depressive symptoms. Although slightly smaller than previously reported estimates, this effect remains clinically meaningful. These results align with previous meta-analyses of mind–body interventions in menopausal women. Innes et al. (59) and Cramer et al. (50) reported reduced depressive symptoms after yoga and Tai Chi, though with smaller effect sizes. By integrating art, music, dance, and Reiki, the present study extends prior evidence and shows broader psychological benefits beyond exercise-based MBTs. MBTs may enhance emotion regulation, promote mindfulness, reduce HPA axis overactivity, and decrease pro-inflammatory cytokines (7, 19). Longer-duration interventions again appeared more effective, and Asian-based studies showed relatively greater effect sizes.

Both somatic and expressive MBTs were beneficial, suggesting that physical engagement and emotional expression each play therapeutic roles in alleviating depressive symptoms. Mindfulness enhances self-awareness and cognitive reappraisal, reducing ruminative thought patterns and stress reactivity. Music therapy evokes positive affect, stimulates reward-related neural pathways, and enhances motivation, thereby counteracting depressive states (45). Dance therapy integrates rhythmic movement and embodied emotion, promoting emotional regulation and social connectedness (60). Pilates, Qigong, and Reiki further contribute to mood stabilization through gentle physical activation, controlled breathing, and energy regulation. These results support the inclusion of expressive MBTs as viable and accessible options for managing menopausal depression, especially in non-clinical or community-based settings.

From a clinical perspective, MBTs can serve as adjunctive or alternative interventions for depressive symptoms in menopausal women. Policymakers should promote access to affordable, evidence-based MBT programs, particularly in resource-limited settings. Future trials should directly compare MBTs to standard treatments, assess long-term efficacy, and investigate biological mechanisms, including hypothalamic–pituitary–adrenal (HPA) axis modulation and neurogenesis (61, 62).

### 4.3 Anxiety

Anxiety symptoms were also significantly reduced following MBTs, the largest pooled effect among all outcomes. These results align with neuroimaging and psychophysiological studies indicating that MBTs attenuate amygdala hyperactivity, enhance cognitive control, and improve vagal tone, thereby reducing anxiety (63, 64). Programs lasting ≥12 weeks were more effective, and interestingly, non-Asian studies showed greater anxiety reductions, possibly due to differences in baseline anxiety severity, cultural perceptions of mental health, or reporting patterns. Regional publication bias and participant characteristics may also play a role. Compared with previous reviews, the current meta-analysis identified a larger reduction in anxiety symptoms. Prior studies Cramer et al. (50) primarily examined yoga or Tai Chi, reporting small-to-moderate effects. The inclusion of non-exercise interventions in this study—particularly art, music, and mindfulness-based therapies—appears to have strengthened the overall effect. The inclusion of diverse MBT modalities in this analysis—particularly mindfulness, Pilates, dance therapy, and Qigong—appears to have strengthened the overall anxiolytic effect. These findings suggest that interventions integrating expressive, meditative, and movement-based components may be more effective for regulating anxiety responses than purely cognitive or physical approaches alone. Consistent with earlier evidence, our subgroup analyses also indicated greater benefits with interventions lasting ≥12 weeks and in Asian populations, suggesting that sustained engagement and cultural alignment may enhance anxiety reduction. However, these findings should be interpreted cautiously, as regional publication bias and population differences could also influence the observed effects.

Several MBTs demonstrated strong anxiolytic effects, with mindfulness, Pilates, Qigong, and dance therapy outperforming other modalities. These approaches emphasize breath regulation, body awareness, and expressive movement, which may enhance emotional regulation through top–down and bottom–up mechanisms. Mindfulness promotes cognitive reappraisal and decreases hyperarousal by strengthening prefrontal control, whereas Pilates and Qigong integrate gentle physical activation with controlled breathing to stabilize autonomic balance. Dance therapy may further support emotional self-regulation and social connectedness through embodied rhythmic expression (65). These mechanisms are consistent with neurophysiological evidence showing that MBTs attenuate amygdala hyperactivity and enhance prefrontal modulation of stress and anxiety responses (12).

In clinical practice, MBTs—particularly mindfulness, Pilates, Qigong, and dance therapy—should be considered viable non-pharmacological options for anxiety management during menopause. These modalities regulate physiological arousal, promote emotional stability, and enhance body–mind integration through relaxation, controlled breathing, and expressive movement. Policy strategies should support MBT instructor training, insurance reimbursement, and public education to improve accessibility and adherence. Future research should prioritize diverse samples, physiological outcome measures (e.g., heart rate variability, cortisol), and examine synergistic effects when combined with conventional therapies (66, 67).

## 5 Limitations

Several limitations should be acknowledged. First, substantial heterogeneity was observed across studies despite the use of random-effects models and subgroup analyses. This may reflect differences in intervention category, intervention duration, frequency, outcome measures, and participant characteristics, limiting the generalizability of pooled estimates.

Second, most trials showed “some concerns,” mainly due to lack of blinding and reliance on self-reported outcomes, which may have inflated effect estimates. Blinding is inherently difficult in MBT research and may increase expectancy bias. To address methodological variability, we used a random-effects model and conducted subgroup analyses. These analyses showed consistent patterns, indicating robustness within the limitations of unblinded and self-reported measures. Future studies should use objective outcomes and blinded assessors to reduce bias.

Third, many trials had small sample sizes, reducing statistical power for subgroup analyses.

Fourth, the included studies used diverse outcome measures. Sleep outcomes were assessed mainly by the PSQI, ISI, and WHI-IRS. Depression and anxiety were measured using validated scales such as the BDI, HADS, DASS-21, and STAI. Although these tools are reliable, their self-reported nature may introduce subjectivity.

Fifth, the broad categorization of MBTs may introduce conceptual heterogeneity, making it difficult to isolate modality-specific effects.

Finally, only peer-reviewed English studies were included. Grey literature and non-English publications were excluded in advance. This may have introduced language and publication bias, which should be considered when interpreting the results.

## 6 Conclusion

This systematic review and meta-analysis provides evidence supporting the potential efficacy of MBTs in improving sleep quality and reducing depressive and anxiety symptoms among perimenopausal and postmenopausal women. Across 18 randomized controlled trials (*n* = 1,572), interventions such as Yoga, Mindfulness-based programs, Pilates, Qigong, Music therapy, Dance therapy, and Reiki demonstrated moderate-to-large beneficial effects. For sleep outcomes, Music therapy yielded the strongest improvements, followed by Mindfulness, Yoga, and Qigong. Subgroup analyses further revealed that Mindfulness, Pilates, Music therapy, Dance therapy, and Reiki produced greater improvements in depression and anxiety than Yoga and Qigong interventions. Moreover, interventions lasting ≥12 weeks and studies conducted in Asian populations showed stronger effects, possibly reflecting differences in intervention type, dosage, and cultural context. Other factors—such as regional publication bias, population characteristics, and adherence differences—may also contribute to these results. While studies from Asia demonstrated particularly pronounced benefits, this may reflect differences in intervention type, duration, or cultural familiarity. However, potential regional publication bias and variations in participant characteristics should also be considered when interpreting these findings. Despite moderate heterogeneity and methodological limitations—including the reliance on self-reported, unblinded outcomes—sensitivity analyses indicated consistent trends across models, suggesting robustness within these constraints.

Given their accessibility, safety, and broad psychosocial benefits, MBTs represent promising non-pharmacological interventions that can complement conventional menopausal care. Expressive and meditative modalities such as Mindfulness, Music, Dance, Qigong, and Reiki may provide additional value through emotional regulation, body awareness, and relaxation. Integrating these approaches into clinical guidelines and community health programs should proceed cautiously until further high-quality RCTs confirm efficacy in midlife women. Future research should optimize intervention protocols, explore underlying physiological mechanisms, and expand evidence across diverse populations. Large, preregistered multicenter trials using standardized MBT designs, objective biomarkers, and long-term follow-up are warranted to strengthen reliability and clarify the sustained effects of these interventions.

## Data Availability

The original contributions presented in the study are included in the article/Supplementary material, further inquiries can be directed to the corresponding author.
